# Ursolic Acid Suppresses Colorectal Cancer Through Autophagy–Lysosomal Degradation of β-Catenin

**DOI:** 10.3390/ijms26136210

**Published:** 2025-06-27

**Authors:** Chung-Ming Lin, Min-Chih Chao, Hsin-Han Chen, Hui-Jye Chen

**Affiliations:** 1 Department of Biotechnology, Ming Chuan University, Taoyuan 33348, Taiwan; cml@mail.mcu.edu.tw; 2 Graduate Institute of Biomedical Sciences, China Medical University, Taichung 40402, Taiwan; bj59420@gmail.com; 3 Division of Plastic and Reconstructive Surgery, Department of Surgery, China Medical University Hospital, Taichung 40402, Taiwan; scapulachenhh@yahoo.com.tw

**Keywords:** colorectal cancer, Wnt signaling, ursolic acid, autophagy, β-catenin

## Abstract

Colorectal cancer remains a leading malignancy. As the aberrant activation of Wnt/β-catenin signaling causes colorectal cancer, Wnt/β-catenin signaling inhibitors are potential candidates for colorectal cancer treatment. Our drug screening platform identified ursolic acid (UA), a triterpenoid with various biological activities, as a potential anticancer drug because it inhibits the T-cell factor (TCF)/β-catenin-mediated transcriptional activity. Here, we discovered that UA inhibited Wnt signaling by reducing the Wnt reporter activity and Wnt target gene expression, leading to a delay in cell cycle progression and the suppression of cell proliferation. Stepwise epistatic analyses suggested that UA functions on β-catenin protein stability in Wnt signaling. Further studies revealed that UA reduced β-catenin protein levels by Western blotting and immunofluorescent staining and induced autophagy by microtubule-associated protein 1 light chain 3 beta (LC3B) punctate staining. The cotreatment with UA and the autophagy inhibitors chloroquine and wortmannin recovered the β-catenin protein levels. Therefore, UA was confirmed to induce β-catenin degradation by the autophagy–lysosomal degradation system through inhibition in the phosphatidylinositol 3-kinase (PI3K)/Ak strain transforming (protein kinase B; AKT)/mammalian target of rapamycin (mTOR) signaling pathway. Our results not only highlight the potential of UA in Wnt-driven colorectal cancer therapy but also provide a workable Wnt signaling termination approach for the treatment of other Wnt-related diseases.

## 1. Introduction

Wnts are secreted lipoglycoproteins that participate in various biological processes, including embryo development, adult cell homeostasis, and stem cell maintenance [1]. In the absence of a Wnt ligand, an Axin complex (or β-catenin-destruction complex), consisting of Axin1, Adenomatous Polyposis Coli (APC), β-catenin, Glycogen synthase kinase-3 beta (GSK3β), and Casein kinase I alpha (CKIα), is formed in the cytoplasm. β-catenin is sequentially phosphorylated by CKIα and GSK3β; phosphorylated β-catenin is then tagged with polyubiquitin and subjected to proteasome degradation. Under this condition, Wnt target genes cannot be activated. By contrast, in the presence of a Wnt ligand, Wnt binds to the extracellular domains of the seven-pass receptor Frizzled and the single-pass coreceptor low-density lipoprotein receptor-related protein 5/6 (LRP5/6). Dishevelled becomes recruited to the cell membrane and binds to Frizzled. The Axin complex is also transported to the cell membrane, and Axin from the complex binds to phosphorylated LRP5/6. Thereafter, β-catenin is stabilized; it then accumulates in the cytoplasm, is translocated to the nucleus, and complexes with TCF/Lymphoid Enhancer Binding Factor (LEF), activating the expression of Wnt target genes such as the *Axin2*, *cyclin D1*, and *c-Myc* genes [2]. An aberration in Wnt signaling results in many cancer types, including colorectal cancer [3]. Therefore, targeting the Wnt/β-catenin pathway may facilitate effective cancer treatment.

Colorectal cancer is the third most common cancer and the second most common cause of cancer-related death worldwide [4]. Several factors contribute to colon carcinogenesis, including environmental factors, chronic inflammation, and genetic predispositions [5]. Environmental factors such as poor diet, smoking, alcohol consumption, and physical inactivity can increase colorectal cancer risk [6]. Chronic inflammation can create a microenvironment contributing to colon tumorigenesis through the production of inflammatory mediators that induce the formation of oxygen radical species that damage DNA, the proliferation of cells, the secretion of metalloproteases that degrade the extracellular matrix for facilitating cancer invasion, and survival factors leading to decreased apoptosis [7]. The accumulation of oncogene and tumor suppressor gene mutations may drive the occurrence of colorectal cancer in a stepwise manner [8,9]. In approximately 80% of patients with sporadic colorectal cancer, the APC gene is mutated; moreover, approximately 50% of patients having colon cancer with wild-type APC harbor mutations in the β-catenin gene [10]. In addition to those in the Wnt/β-catenin signaling genes, mutations in the RAS-RAF-mitogen-activated protein kinase (MAPK) pathway genes, such as v-raf murine sarcoma viral oncogene homolog B1 (*BRAF*) and the Kirsten rat sarcoma viral oncogene homolog (*KRAS*), also facilitate the disease progression [11]. Other mechanisms, such as chromosomal instability, microsatellite instability, and the CpG island methylator phenotype, are also involved [12]. Different stages of colorectal cancers are treated using different strategies. In patients with stage I, II, or III disease, surgery is the treatment of choice. Adjuvant chemotherapy and radiotherapy are mainly prescribed to patients with stage II and III cancer, whereas patients with metastasis and those at stage IV—who cannot be treated with surgery, radiotherapy, or chemotherapy—are typically offered palliative therapy [13,14,15]. In addition, combination therapy and antibodies that target the epidermal growth factor receptor (EGFR) and vascular endothelial growth factor receptor (VEGFR) are used to treat selected patients with colorectal cancer [16,17]. Despite these advancements, more workable approaches for colorectal cancer treatment are required.

Ursolic acid (UA; i.e., 3β-3-hydroxy-urs-12-ene-28-oic-acid) is a pentacyclic triterpenoid found in many fruits and vegetables [18]. This phytochemical possesses a broad range of biological activities, including anti-inflammatory, antimicrobial, antioxidant, immunomodulatory, hepatoprotective, cardioprotective, antihyperlipidemic, hypoglycemic, skin-protective, chemopreventive, and antitumor activities [19]. Moreover, it can increase muscle growth and promote fat loss [20]. UA demonstrates activity against various types of cancers, including breast, colorectal, liver, lung, gallbladder, gastric, prostate, and oral cancers [21]. The mechanism underlying its anticancer activity involves the inhibition of cell invasion, metastasis, epithelial–mesenchymal transition, inflammation, cell cycle progression, apoptosis induction, decreased chemotherapy and radiotherapy resistance, and immunoregulation [21]. Because of its low cytotoxicity [21], UA may be an ideal drug for cancer treatment.

Autophagy is a mechanism that delivers damaged cellular components, such as proteins and organelles, to the lysosome for degradation, and this process provides energy and recycled materials for anabolic processes. It is a highly complicated evolutionary biological process that can be activated by several stress conditions, such as reactive oxygen species (ROS), hypoxia, chemotherapy, and radiotherapy [22]. Autophagy can be divided into several consecutive steps: initiation and elongation, closure, maturation, and degradation. In general, autophagy is initiated through the activation of the serine/threonine kinase unc-51-like autophagy activating kinase (ULK1) complex, which then activates the class III phosphatidylinositol 3-kinase (PI3K) complex to induce phosphatidylinositol 3-phosphate (PI3P) formation at the nucleation site of the phagophore originating from the endoplasmic reticulum (ER). The cysteine protease Autophagy Related 4B cysteine peptidase (ATG4B) converts ProLC3B to LC3B-I, which then conjugates with phosphatidylethanolamine (PE) to form LC3B-II through two ubiquitin-like conjugation systems. LC3B-II is then inserted into the phagophore membranes. The phagophore expands and engulfs cargos. Next, the phagophore membrane closes to form an autophagosome. An autophagosome then becomes fused with a lysosome to form an autolysosome. The cargos are digested inside autolysosomes, and the degraded materials are recycled for biogenesis [23,24].

In this study, we used a series of molecular, cellular, and biochemical analyses to examine the effects and underlying mechanism of UA on colorectal cancer cells, and we discovered that UA can suppress cell proliferation and delay cell cycle progression in colorectal cancer cells through the Wnt signaling inhibition. Further evidence demonstrated that UA can downregulate the Wnt signaling critical component β-catenin by the autophagy–lysosomal degradation system through the inhibition of the PI3K/AKT/mTOR pathway. Our studies highlight the potential application of UA for the treatment of colorectal cancer.

## 2. Results

### 2.1. UA Reduces Wnt/β-Catenin Signaling in Wnt-Stimulated P19 Cells and COS-7 Cells

Wnt/β-catenin signaling is vital in different biological processes, and its deregulation results in human diseases, including various cancers [25]. Therefore, a chemical that can suppress aberrant Wnt/β-catenin signaling is highly likely to become a potential therapeutic drug. To this purpose, UA was screened out for its ability to inhibit TCF/β-catenin-mediated transcriptional activity.

**Figure 1:**
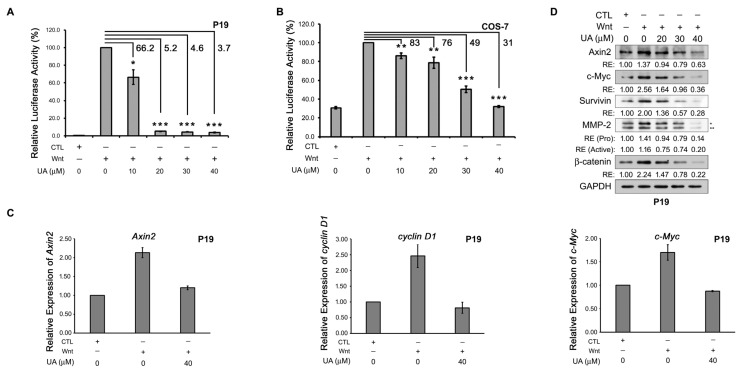
UA inhibits Wnt signaling in Wnt-challenged cells. (**A**) UA inhibited Wnt signaling in Wnt-stimulated P19 cells. P19 cells were transfected with Wnt reporter pGL3-OT and normalization vector pTK-Renilla (dual reporters), treated with control-conditioned medium (CTL), Wnt-3a-conditioned medium (Wnt), or Wnt-3a-conditioned medium with UA at different concentrations (0, 10, 20, 30, or 40 µM). After 16 h, cell lysates were collected for dual luciferase activity assay. Three replicates were assessed for each treatment. Ratio of firefly luciferase activity to Renilla luciferase activity at 0 µM UA under Wnt stimulation was set at 100%; ratios for all other treatments were calculated accordingly. (**B**) UA inhibited Wnt signaling in Wnt-stimulated COS-7 cells. COS-7 cells were treated as indicated in (**A**); cell lysate was collected for dual luciferase activity assay. Three replicates were assessed for each treatment. (**C**) UA reduced mRNA expression of Wnt target genes in Wnt-stimulated P19 cells. P19 cells were treated with control-conditioned medium (CTL), Wnt-3a-conditioned medium (Wnt), or Wnt-3a-conditioned medium with UA (40 µM). After 16 h, cells were collected for RNA isolation, cDNA synthesis, and quantitative PCR amplification on *Axin2* (left panel), *cyclin D1* (middle panel), and *c-Myc* (right panel) genes. Gene expression with control-conditioned medium was set as 1.0, and gene expression with other treatments was calculated accordingly. (**D**) UA suppressed protein expression of Wnt target genes and β-catenin in Wnt-stimulated P19 cells. P19 cells were treated with control-conditioned medium (CTL), Wnt-3a-conditioned medium (Wnt), or Wnt-3a-conditioned medium with UA at different concentrations (0, 20, 30, or 40 µM). Cell lysates were obtained for Western blotting analyses of Axin2, c-Myc, survivin, MMP2, and β-catenin. MMP2 antibody was used to detect both pro-MMP2 (*, 72 kDa) and active MMP2 (**, 66 kDa). GAPDH was loading control. *, *p* < 0.05. **, *p* < 0.01. ***, *p* < 0.001. RE, relative expression. Numbers are percentages of dual luciferase activity relative to that of 0 μM UA with Wnt.

To confirm the Wnt-inhibiting activity of UA, P19 cells were transfected with the Wnt reporter plasmid pGL3-OT and the normalization plasmid pTK-Renilla and then treated with a control-conditioned or a Wnt-3a-conditioned medium containing different concentrations of UA. Being embryonic carcinoma cells with pluripotent properties, P19 cells can be differentiated into endoderm-, mesoderm-, and ectoderm-like cells through treatment with different reagents [26]. Moreover, P19 cells demonstrate a favorable response to Wnt treatment [27,28]. As shown in Figure 1A, treatment with the Wnt-3a-conditioned medium induced strong TCF/β-catenin-mediated transcriptional activity in P19 cells compared with treatment with the control-conditioned medium. The UA treatment reduced the Wnt-induced luciferase activity in the P19 cells in a concentration-dependent manner. The UA treatment also suppressed the Wnt-induced luciferase activity in the COS-7 cells (Figure 1B). Because UA can inhibit Wnt reporter activity, we examined the effects of UA on the mRNA expression of the Wnt target genes in the Wnt-treated P19 cells through RT-qPCR. The Wnt treatment was noted to increase the mRNA expression of the Wnt target genes, including the *Axin2*, *cyclin D1*, and *c-Myc* genes. However, the UA treatment reduced the Wnt-induced mRNA expression of these Wnt target genes in the P19 cells (Figure 1C). The UA treatment also reduced the protein levels of the Wnt-induced β-catenin and Wnt target genes, including the Axin2, c-Myc, survivin, and MMP2 genes in the P19 cells (Figure 1D). These data suggested that UA can inhibit Wnt/β-catenin signaling in Wnt-stimulating cells.

### 2.2. UA Reduces Wnt/β-Catenin Signaling in Colorectal Cancer Cells

**Figure 2:**
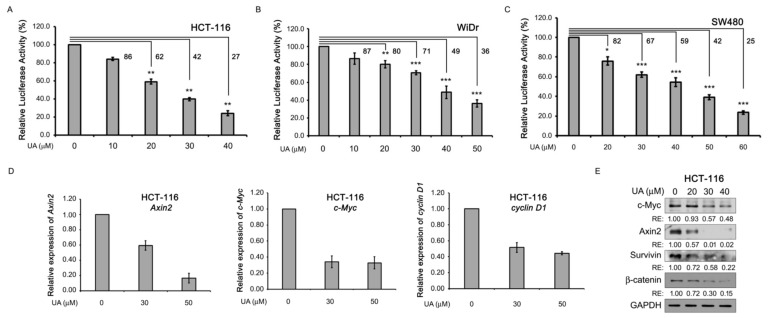
UA inhibits Wnt signaling in colorectal cancer cells. (**A**) UA reduced TCF/β-catenin-mediated luciferase activity in HCT-116 cells. HCT-116 cells were transfected with dual reporters and treated with UA at different concentrations (0–40 µM) for 22 h. Cell lysates were obtained for dual luciferase activity assay. Activity with 0 µM UA was set at 100%, and activities with all other treatments were calculated accordingly. (**B**) UA suppressed TCF/β-catenin-mediated luciferase activity in WiDr cells. WiDr cells were transfected with dual reporters and treated with UA at different concentrations (0–50 µM) for 22 h. Cell lysates were obtained for dual luciferase activity assay. (**C**) UA decreased TCF/β-catenin-mediated luciferase activity in SW480 cells. SW480 cells were transfected with dual reporters and treated with UA at different concentrations (0–60 µM). Cell lysates were obtained for dual luciferase activity assay. (**D**) UA suppressed mRNA expression of Wnt target genes in HCT-116 cells. HCT-116 cells were treated with UA at different concentrations (0, 30, or 50 µM) for 22 h. Cells were collected for RNA isolation, cDNA synthesis, and quantitative PCR amplification on *Axin2* (left panel), *c-Myc* (middle panel), and *cyclin D1* (right panel) genes. Gene expression at 0 µM UA was set at 1.0, and that for all other treatments was calculated accordingly. (**E**) UA reduced protein levels of Wnt target genes and β-catenin in HCT-116 cells. HCT-116 cells were treated with UA at different concentrations (0, 20, 30, or 40 µM) for 22 h. Cells were collected for Western blotting analyses of c-Myc, Axin2, survivin, and β-catenin. GAPDH was loading control. *, *p* < 0.05. **, *p* < 0.01. ***, *p* < 0.001. RE: relative expression. Numbers are percentages of dual luciferase activity relative to that of 0 μM UA.

Because UA inhibits Wnt signaling in Wnt-stimulated P19 and COS-7 cells (Figure 1), we examined the effects of UA on Wnt-driven colorectal cancer cell lines, including HCT-116, WiDr, and SW480 cells. These cells can maintain active Wnt signaling because mutations in either the β-catenin (HCT-116 cells) or APC (WiDr and SW480 cells) gene [29,30] lead to an aberrant β-catenin accumulation in the cytoplasm, followed by the activation of β-catenin-mediated downstream Wnt signaling. Thus, HCT-116, WiDr, and SW480 colorectal cancer cells were transfected with Wnt reporters, treated with UA at different concentrations, and subjected to a dual luciferase activity assay. As shown in Figure 2A–C, the UA treatment inhibited the TCF/β-catenin-mediated transcriptional activity in the HCT-116 (Figure 2A), WiDr (Figure 2B), and SW480 (Figure 2C) cells. Next, the effects of UA on the expression of the Wnt target genes in the HCT-116 cells were examined. The UA treatment decreased the mRNA expression of the Wnt target genes, including the *Axin2*, *c-Myc*, and *cyclin D1* genes, through RT-qPCR analyses (Figure 2D). The UA treatment decreased the protein levels of β-catenin in a concentration-dependent manner and suppressed the protein expression of the Wnt target genes, including the c-Myc, Axin2, and survivin genes, through Western blotting analyses (Figure 2E). These data indicated that in Wnt-driven colorectal cancer cells, UA can inhibit Wnt signaling.

### 2.3. UA Suppresses the Proliferation of Wnt-Stimulated Cells and Colorectal Cancer Cells

**Figure 3:**
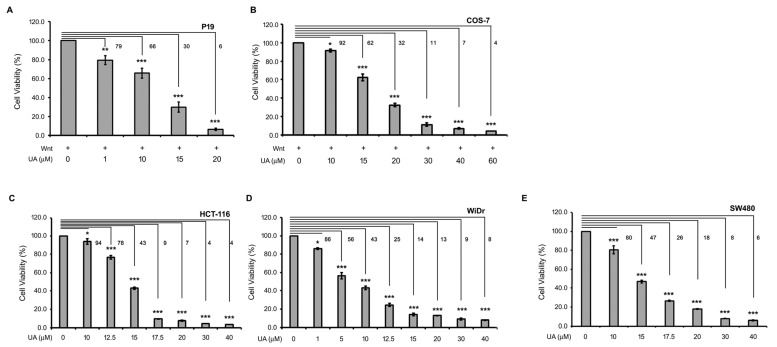
UA suppresses proliferation in Wnt-stimulated cells and colorectal cancer cells. (**A**) UA reduced Wnt-stimulated P19 cell viability. P19 cells were treated with Wnt-3a-conditioned medium (Wnt) with UA at different concentrations (0, 1, 10, 15, or 20 µM). Cell viability was analyzed using MTT assay. Values for cells treated with Wnt-3a-conditioned medium with 0 µM UA were set at 100%, and values for other treatments were calculated accordingly. (**B**) UA reduced Wnt-stimulated COS-7 cell viability. COS-7 cells were treated with Wnt-3a-conditioned medium (Wnt) or Wnt-3a-conditioned medium with UA at different concentrations (0, 10, 15, 20, 30, 40, or 60 µM). Cell viability was analyzed using MTT assay. (**C**–**E**) UA reduced colorectal cancer cell viability. HCT-116 (**C**), WiDr (**D**), and SW480 (**E**) cells were treated with UA at different concentrations (0–40 µM) for 22 h for MTT assay. Values for cells treated with 0 µM UA were set at 100%, and values for other treatments were calculated accordingly. Three replicates were assessed for each treatment. *, *p* < 0.05. **, *p* < 0.01. ***, *p* < 0.001. Numbers are percentages of cell viability relative to that of 0 μM UA.

Wnt/β-catenin signaling is important for cell proliferation [31]. Therefore, the effects of UA on cell viability were evaluated. P19 cells were treated with a Wnt-3a-conditioned medium with or without UA at different concentrations. Next, the MTT assay was used to examine the cell viability. As shown in Figure 3A, the UA treatment reduced the cell viability in a concentration-dependent manner. The half-maximal inhibitory concentration (IC_50_) of UA for the Wnt-stimulated P19 cells was 10.83 ± 0.68 µM. The UA treatment also repressed the Wnt-stimulated COS-7 cell proliferation (Figure 3B), with an IC_50_ of 18.35 ± 0.07 µM. Because UA reduced the Wnt-stimulated cell viability, we examined the effects of UA on the cell proliferation of the Wnt-driven colorectal cancer cells. HCT-116 cells have mutations in the β-catenin gene, whereas SW480 and WiDr cells harbor truncation mutations in the APC gene. These mutations render the stabilization of β-catenin in cells, leading to β-catenin-mediated downstream Wnt signaling [29]. The UA treatment reduced the HCT-116, WiDr, and SW480 cell viability in a concentration-dependent manner, with IC_50_ values of 14.26 ± 0.05, 7.96 ± 0.05, and 14.61 ± 0.28 µM, respectively (Figure 3C–E). These data indicate that UA can reduce the viability of not only Wnt-stimulated P19 and COS-7 cells but also colorectal cancer cells.

**Figure 4:**
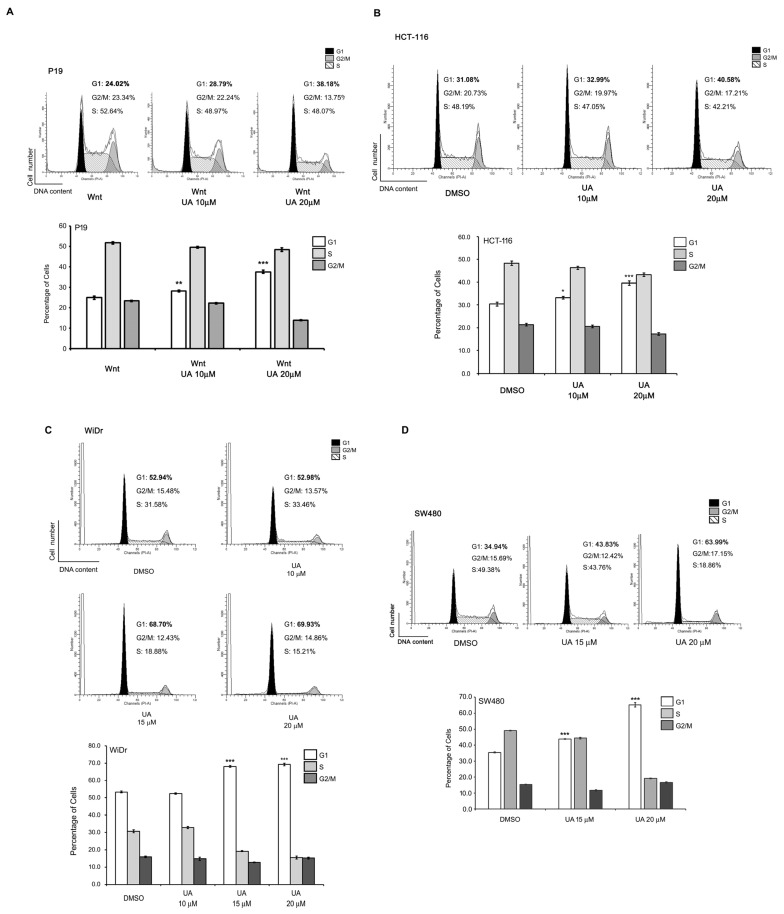
UA delays the cell cycle progression in the G1 phase in Wnt-stimulated P19 and colorectal cancer cells. (**A**) UA arrested the cell cycle in the G1 phase in the Wnt-stimulated P19 cells. The P19 cells were treated with a Wnt-3a-conditioned medium with 0, 10, or 20 µM UA. Next, the cells were stained with propidium iodide and fixed, and their cell cycle progression was analyzed through flow cytometry (Upper panel). The data (% of cells in each phase) are represented by the means ± standard deviations from three replicates (Lower panel). (**B**–**D**) UA delayed the cell cycle progression in the G1 phase in colorectal cancer cells. The HCT-116 cells (**B**) were treated with 0, 10, or 20 µM UA; the WiDr cells (**C**) with 0, 10, 15, or 20 µM UA; and the SW480 cells (**D**) with 0, 15, or 20 µM UA. The cells were stained and then analyzed through flow cytometry. *, *p* < 0.05 versus G1 at DMSO. **, *p* < 0.01 versus G1 at DMSO or G1 at Wnt. ***, *p* < 0.001 versus G1 at DMSO or G1 at Wnt.

Because UA can suppress cell viability, we examined whether UA inhibits the cell cycle progression in Wnt-stimulated P19 cells and colorectal cancer cells. P19 cells were treated with UA at different concentrations in a Wnt-3a-condtioned medium for flow cytometry analysis. The results demonstrated that the UA treatment arrested the cell cycle in the G1 phase (Figure 4A). Next, the effects of the UA treatment on the cell cycle progression in the Wnt-driven HCT-116, WiDr, and SW480 cells were assessed through flow cytometry. The results demonstrated that the UA treatment also suppressed the cell cycle in the G1 phase in colorectal cancer cells (Figure 4B–D). These data suggest that UA can inhibit proliferation in Wnt-stimulated cells and colorectal cancer cells by suppressing cell cycle progression.

### 2.4. UA Downregulates β-Catenin in Wnt Signaling

**Figure 5:**
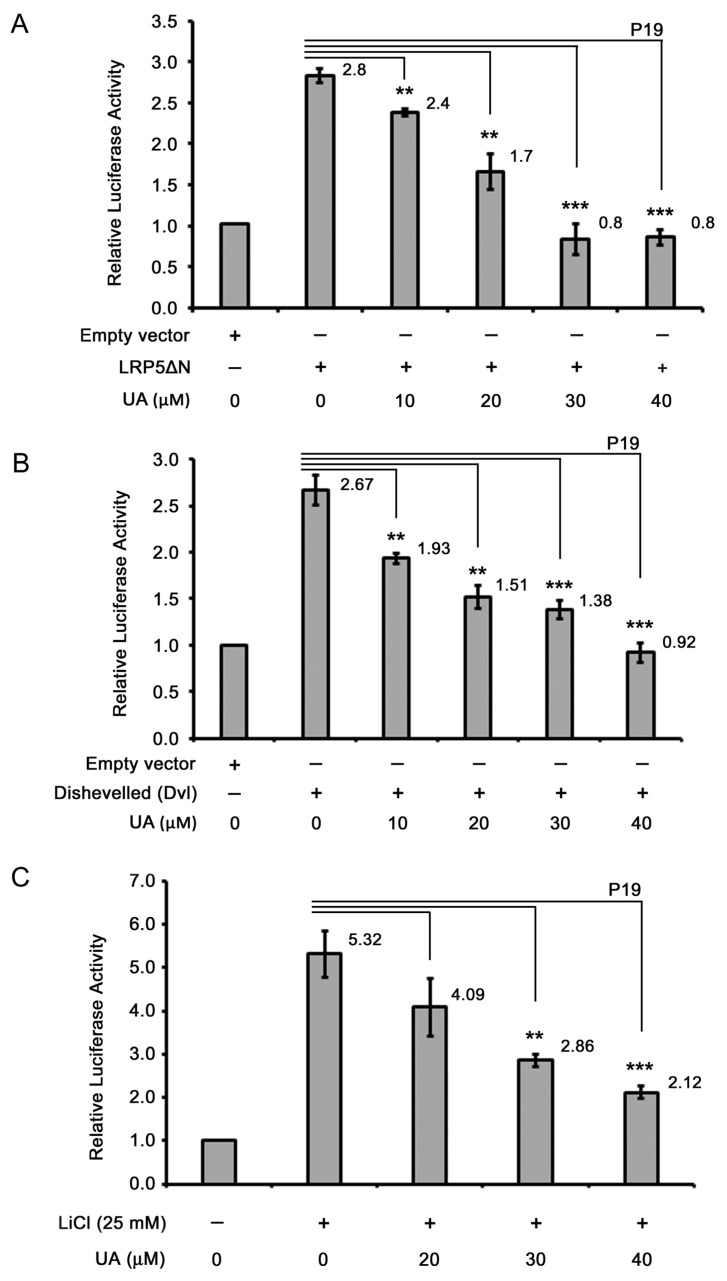
UA acts on either GSK3β or cellular targets downstream of GSK3β in the Wnt pathway. (**A**) UA acted on LRP5 or downstream of LRP5 in the Wnt pathway. P19 cells were transfected with pLRP5△N or an empty vector, as well as pGL3-OT and pTK-Renilla, and then treated with UA at different concentrations (0–40 µM) for 16 h. The cell lysates were collected for a dual luciferase activity assay. (**B**) UA acts on Dishevelled or downstream of Dishevelled in the Wnt pathway. P19 cells were transfected with a Dishevelled-2 plasmid or an empty vector, as well as pGL3-OT and pTK-Renilla, and then treated with UA at different concentrations (0–40 µM) for 16 h. The cell lysates were collected for a dual luciferase activity assay. (**C**) UA acted on GSK3β or downstream of GSK3β in the Wnt pathway. P19 cells were transfected with pGL3-OT and pTK-Renilla and then treated with 25 mM LiCl and UA at different concentrations (0–40 µM) for 16 h. The cell lysates were collected for a dual luciferase activity assay. The values for cells treated with 0 µM UA with no LiCl were set at 1.0, and those for all other treatments were calculated accordingly. Three replicates were assessed for each treatment. **, *p* < 0.01. ***, *p* < 0.001. The numbers are dual luciferase activities relative to 0 μM UA with an empty vector or 0 μM UA with no LiCl.

As UA can inhibit Wnt signaling, the cellular targets of UA in the Wnt/β-catenin pathway were assessed. First, the effects of UA on proteins upstream or downstream of LRP5/6, the coreceptor of Wnt signaling, were examined. To this end, pLRP5△N, a cDNA encoding LRP5 deficient of the N-terminal domain was constructed into the expression vector pcDNA3.1-HA(3) with an in-frame HA-tag [32], was used. The ectopically expressed dominant-active LRP5△N constitutively activates the LRP5-mediated downstream Wnt signaling. The P19 cells were cotransfected with pLRP5△N, pGL3-OT, and pTK-Renilla; the cells were then treated with UA at different concentrations and were analyzed for their dual luciferase activity. Compared with the treatment with an empty vector, the LRP5△N overexpression led to an increase in the β-catenin/TCF-mediated transcriptional activity. The UA treatment led to a decrease in the LRP5△N-induced Wnt signaling in a concentration-dependent manner (Figure 5A), suggesting that UA acts on either LRP5 or downstream of LRP5 in the Wnt pathway.

We then examined whether UA targets proteins upstream or downstream of Dishevelled, a signal transducer in Wnt signaling [33]. Dishevelled overexpression can activate Wnt signaling downstream of Dishevelled [34]. To assess the effects of UA on Dishevelled-mediated downstream Wnt signaling, P19 cells were transfected with cDNA encoding Dishevelled-2 together with dual reporters, and the cells were treated with UA at different concentrations; the dual luciferase activity of the cells was then analyzed. The Dishevelled expression induced an increase in the Wnt signaling compared to that of the empty vector. The UA treatment reduced the Dishevelled-induced Wnt signaling in a concentration-dependent manner (Figure 5B), suggesting that UA acts on Dishevelled or downstream of Dishevelled in the Wnt pathway.

**Figure 6:**
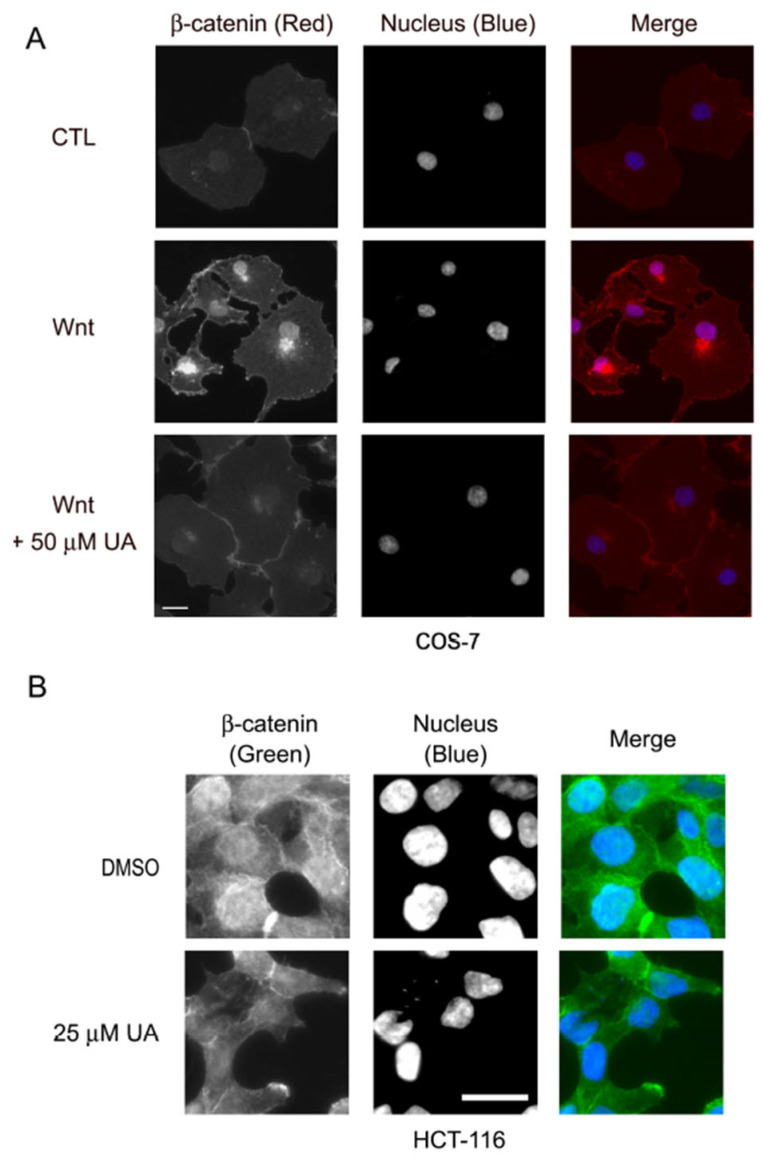
UA reduces β-catenin protein levels in Wnt-stimulated COS-7 and HCT-116 cells by immunofluorescence staining. (**A**) COS-7 cells were treated with control-conditioned medium (CTL), Wnt-3a-conditioned medium (Wnt), or Wnt-3a-conditioned medium with 50 µM UA for 16 h, fixed, and stained for β-catenin (red). Nuclei were stained using DAPI (blue). (**B**) HCT-116 cells were either treated with carrier reagent (DMSO) or with 25 µM UA for 22 h, fixed, and stained for β-catenin (green). Nuclei were stained using DAPI (blue). Scale bar, 20 µm.

We next examined whether UA functions upstream or downstream of GSK3β. GSK3β, a serine/threonine kinase, can phosphorylate β-catenin for the subsequent ubiquitination and proteasomal degradation [35]. The inhibition of GSK3β kinase activity by using the GSKβ inhibitor LiCl leads to β-catenin stabilization, followed by the activation of β-catenin-mediated downstream Wnt signaling. Therefore, in this study, P19 cells were transfected with dual reporters, treated with 25 mM LiCl and UA at different concentrations, and assayed for dual luciferase activity. The results indicated that the LiCl treatment elicited TCF/β-catenin-mediated transcriptional activity, whereas the UA treatment inhibited the LiCl-induced Wnt signaling; therefore, UA may exert its effects on GSK3β itself or cellular targets downstream of GSK3β in the Wnt pathway (Figure 5C). Given that GSK3β can regulate β-catenin stability, we examined the effects of UA on β-catenin stability. To achieve this, P19 cells were treated with a control-conditioned medium or Wnt-conditioned medium with or without UA at different concentrations and were then assessed through Western blotting. As shown in Figure 1D (panel 5), compared with the control-conditioned medium, the Wnt-conditioned medium induced an increase in the β-catenin expression. In the P19 cells, the UA treatment reduced the protein levels of β-catenin in a concentration-dependent manner. The treatment with UA also reduced the protein levels of β-catenin in the HCT-116 cells (Figure 2E, panel 4). The immunofluorescence staining results also revealed that the levels of both cytoplasmic and nuclear β-catenin decreased after the treatment with UA in the Wnt-stimulated COS-7 cells (Figure 6A) and HCT-116 cells (Figure 6B). Therefore, UA may downregulate β-catenin in Wnt signaling.

### 2.5. UA Induces Autophagy–Lysosomal Degradation of β-Catenin in Wnt Signaling

**Figure 7:**
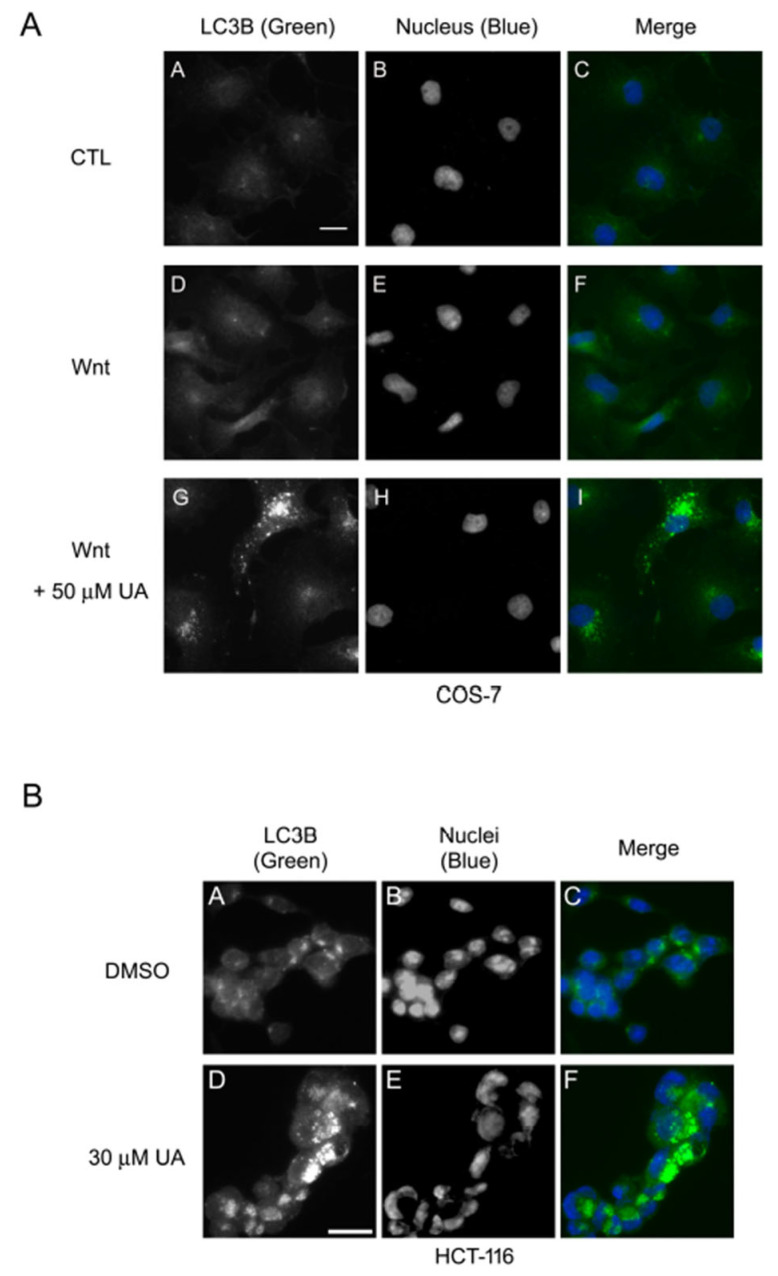
UA induces autophagosome formation in Wnt-stimulated COS-7 and HCT-116 cells. (**A**) UA-induced autophagosome formation in Wnt-stimulated COS-7 cells. COS-7 cells were treated with control-conditioned medium (CTL), Wnt-3a-conditioned medium (Wnt), or Wnt-3a-conditioned medium with 50 µM UA for 16 h. Cells were then fixed, permeabilized, and stained for LC3B (green). Nuclei were stained using DAPI (blue). A–C, treatment with control-conditioned medium. D–F, treatment with Wnt3a-conditioned medium. G–I, treatment with 50 μM UA in Wnt3a-conditioned medium. (**B**) UA-induced autophagosome formation in HCT-116 cells. HCT-116 cells were either treated with carrier reagent (DMSO) or with 30 µM UA for 22 h. Cells were then fixed, permeabilized, and stained for LC3B (green). Nuclei were stained using DAPI (blue). A–C, treatment with carrier reagent. D–F, treatment with 30 μM UA. Scale bar, 20 µm.

The ubiquitin–proteasomal, autophagy–lysosomal, and calpain degradation systems are the three main protein degradation systems in cells [36]. HCT-116 cells harbor β-catenin mutations, which prevent the ubiquitin–proteasomal degradation of β-catenin. The aforementioned data demonstrated that UA can reduce the levels of mutated β-catenin in HCT-116 cells, bypassing the possible involvement of the ubiquitin–proteasomal degradation system in the UA-mediated β-catenin degradation. The UA treatment also induced punctate staining of LC3B in the cytoplasm in both Wnt-stimulated COS-7 cells (Figure 7A) and HCT-116 cells (Figure 7B). Thus, UA may induce autophagosome formation under Wnt conditioning.

**Figure 8:**
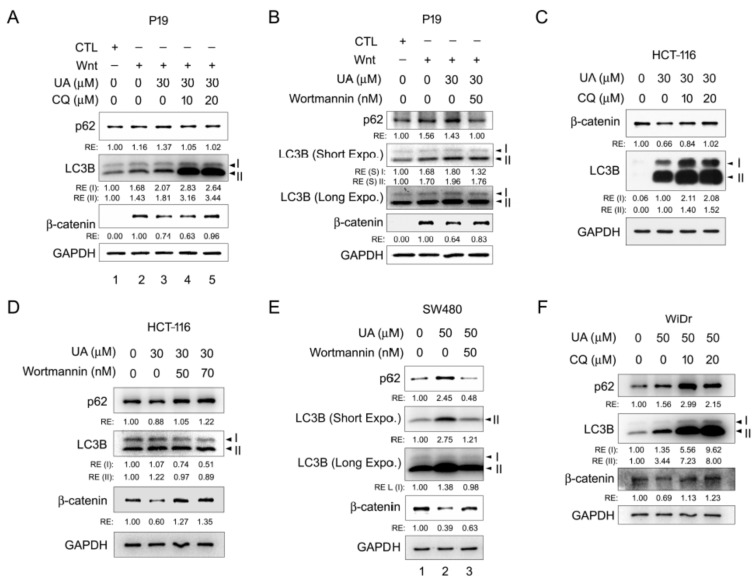
Autophagy inhibition compromises UA-induced β-catenin degradation in Wnt signaling. (**A**) Chloroquine inhibited β-catenin degradation induced by UA in Wnt signaling in P19 cells. P19 cells were pretreated with chloroquine (CQ; 0, 10, or 20 µM) for 2 h and then treated with control-conditioned medium (CTL), Wnt-3a-conditioned medium (Wnt), or Wnt-3a-conditioned medium with UA (30 µM) and chloroquine (0, 10, or 20 µM) for 16 h. Cells were collected for Western blotting analyses of p62, LC3B, and β-catenin proteins. GAPDH was loading control. (**B**) Wortmannin inhibited β-catenin degradation induced by UA in Wnt-treated P19 cells. P19 cells were pretreated with wortmannin (0 or 50 nM) for 2 h and then treated with control-conditioned medium (CTL), Wnt-3a-conditioned medium (Wnt), or Wnt-3a-conditioned medium with 30 µM UA and wortmannin (0 or 50 nM) for 16 h. Cells were collected for Western blotting analyses of p62, LC3B, and β-catenin. Short Expo., short exposure. Long Expo., long exposure. (**C**) UA-induced downregulation of β-catenin was suppressed by chloroquine in HCT-116 cells. HCT-116 cells were pretreated with chloroquine (0, 10, or 20 µM) for 2 h and then treated with UA (0 or 30 µM) or with both UA (30 µM) and chloroquine (10 or 20 µM) for 22 h. Cells were collected for Western blotting analyses of β-catenin and LC3B. GAPDH was loading control. (**D**) UA-induced degradation of β-catenin was abolished by wortmannin in HCT-116 cells. HCT-116 cells were pretreated with wortmannin (50 or 70 nM) for 2 h and then treated with UA (0 or 30 µM) or with both UA (30 µM) and wortmannin (0, 50, or 70 nM) for 22 h. Cells were collected for Western blotting analyses of p62, LC3B, and β-catenin. GAPDH was loading control. (**E**) Wortmannin inhibited β-catenin degradation elicited by UA in SW480 cells. SW480 cells were pretreated with wortmannin (0 or 50 nM) for 2 h and then treated with vehicle, UA (50 µM), or both wortmannin (50 nM) and UA (50 µM) for 22 h. Cells were collected for Western blotting analyses of p62, LC3B, and β-catenin. GAPDH was loading control. (**F**) Chloroquine repressed UA-induced degradation of β-catenin in WiDr cells. Cells were pretreated with chloroquine (CQ; 0, 10, or 20 µM) for 2 h and then treated with UA (0 or 50 µM), or with both UA (50 µM) and chloroquine (10 or 20 µM) for 22 h. Cells were collected for Western blotting analyses with p62, LC3B, and β-catenin antibodies. GAPDH was loading control. RE: relative expression. LC3B-I, an unconjugated form. LC3B-II, a phosphatidylethanolamine-conjugated form.

We then examined the possible involvement of the autophagy–lysosomal degradation system in the UA-induced β-catenin degradation under Wnt conditioning. P19 cells were incubated in a control-conditioned medium or Wnt-conditioned medium with or without UA and chloroquine at different concentrations and then assessed through Western blotting. Chloroquine is an autophagy inhibitor that can interfere with the autophagosome–lysosome fusion [37]. The treatment with the Wnt-conditioned medium induced β-catenin stabilization, whereas the treatment with UA in the Wnt-conditioned medium reduced the β-catenin protein levels. Further treatment with chloroquine led to the recovery of β-catenin protein levels. The expression of the autophagy protein markers LC3B and p62/SQSTM1 [38] also fluctuated concomitantly (Figure 8A). We also evaluated the effects of wortmannin on β-catenin stability in the Wnt-treated P19 cells. Wortmannin is an inhibitor to PI3K that is required for autophagy [39]. Wortmannin increased the β-catenin protein levels, which were downregulated by UA (Figure 8B). Similar results were observed for the Wnt-driven HCT-116, SW480, and WiDr cells (Figure 8C–F). Taken together, these results indicated that the autophagy–lysosomal degradation system is responsible for the UA-mediated β-catenin downregulation during Wnt signal activation.

### 2.6. The PI3K/AKT/mTOR/ULK Pathway Is Involved in UA-Induced Autophagy–Lysosomal Degradation of β-Catenin in Wnt Signaling

**Figure 9:**
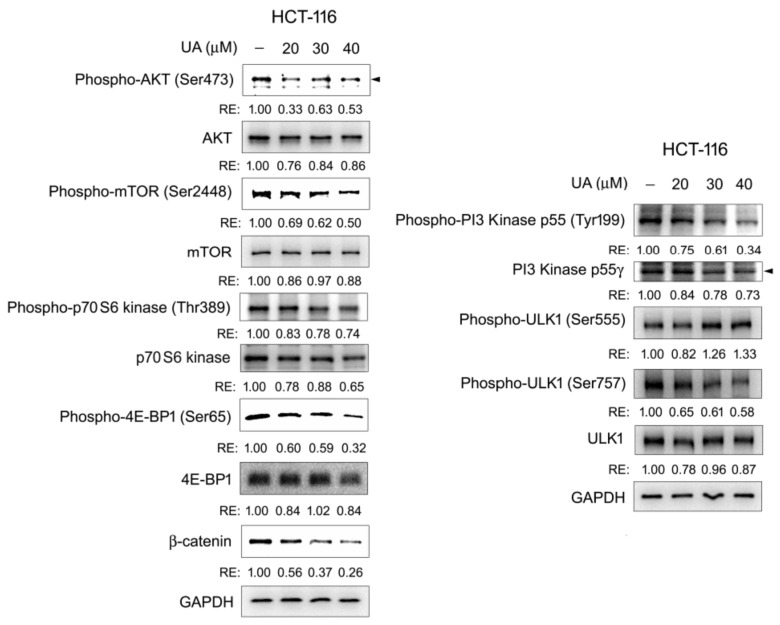
UA induces autophagic degradation of β-catenin through inhibition in PI3K/AKT/mTOR pathway. HCT-116 cells were treated with UA at different concentrations (0, 20, 30, or 40 µM) for 22 h. Western blots for mTOR, phosphorylated mTOR (Ser2448), AKT, phosphorylated AKT (Ser473), PI3 kinase p55γ, phosphorylated PI3 kinase p55 (Tyr199), ULK1, phosphorylated ULK1 (Ser757), phosphorylated ULK1 (Ser555), 4E-BP1, phosphorylated 4E-BP1 (Ser65), p70 S6 kinase, phosphorylated p70 S6 kinase (Thr389), and β-catenin proteins were obtained. GAPDH was loading control. Left arrowhead denotes the detected phosphorylated form of AKT (Ser473). Right arrowhead denotes the detected the PI3 kinase p55γ. RE: relative expression.

The PI3K/AKT/mTOR/ULK pathway participates in autophagy [40]. When a ligand (such as a growth factor) binds to the membrane receptor, PI3K becomes activated and catalyzes the formation of phosphatidylinositol (3,4,5) trisphosphate (PIP3), which recruits AKT to the cell membrane. AKT is then phosphorylated at threonine 308 and serine 473 for its full activation, and it subsequently activates mTORC1, an mTOR complex. Next, mTOR activates downstream substrates, including 4E-BP1 and p70 S6 kinase, promoting cell growth and inhibiting autophagy [41]. The inhibition of this pathway results in mTOR activity suppression, followed by autophagy initiation. The suppression of mTOR activity reduces the phosphorylation of ULK1 at serine 757, which increases AMP-activated protein kinase (AMPK) binding to ULK1 and ULK1 phosphorylation at serine 555 by AMPK, leading to autophagy initiation [42]. To understand the mechanism underlying the UA-elicited β-catenin degradation, Wnt-driven HCT-116 cells were treated with UA at different concentrations, and proteins in the signaling pathways, including AKT, phosphorylated AKT, mTOR, phosphorylated mTOR, p70 S6 kinase, phosphorylated p70 S6 kinase, 4E-BP1, phosphorylated 4E-BP1, ULK1, phosphorylated ULK1, PI3 kinase p55γ, and phosphorylated PI3 kinase p55, were examined through Western blotting. As illustrated in Figure 9, UA reduced β-catenin protein levels in a concentration-dependent manner. UA did not affect or had a minimal effect on the AKT, mTOR, p70 S6 kinase, 4E-BP1, and ULK1 protein levels. However, UA reduced the protein levels of phosphorylated AKT (Ser473), phosphorylated mTOR (Ser2448), phosphorylated p70 S6 kinase (Thr389), and phosphorylated 4E-BP1 (Ser65). The protein levels of PI3 kinase p55γ and phosphorylated PI3 kinase p55 (Tyr199) were decreased. In addition, phosphorylated ULK1 (Ser555) levels increased, but phosphorylated ULK1 (Ser757) levels decreased in a concentration-dependent manner. These data indicated that the PI3K/AKT/mTOR pathway inhibition is essential for the UA-induced autophagy–lysosomal degradation of β-catenin involved in Wnt signaling.

## 3. Discussion

Ursolic acid is a natural triterpenoid compound with several beneficial effects, including antioxidant, anti-inflammatory, antimicrobial, and anticancer activities [43]. UA displays anticancer activities both in vitro and in vivo in multiple cancer types, such as breast cancer [44], skin cancer [45], liver cancer [46], pancreatic cancer [47], cervical cancer [48], ovarian cancer [49], and bladder cancer [50], through the inhibition of cell proliferation [51], the induction of cell cycle arrest [52], and the promotion of apoptosis [44]. In this study, the effects of UA were evaluated in Wnt-stimulated P19 cells (embryonic carcinoma cells) and COS-7 cells, as well as in Wnt-driven HCT-116, WiDr, and SW480 colorectal cancer cells. The results indicated that UA inhibited the TCF/β-catenin-dependent luciferase activity in the Wnt-stimulated cells (Figure 1A,B) as well as in the Wnt-driven colorectal cancer cells (Figure 2A–C). Moreover, UA can suppress the mRNA and protein expression of Wnt target genes in Wnt-stimulated P19 cells (Figure 1C,D) and HCT-116 cells (Figure 2D,E). These data suggest that UA can inhibit Wnt/β-catenin signaling. Further analyses of the effects of UA on Wnt-dependent biological activities demonstrated that UA suppresses cell proliferation and delays the cell cycle in the G1 phase in Wnt-stimulated P19 and COS-7 cells as well as in HCT-116, WiDr, and SW480 cells (Figure 3 and Figure 4). Compared with the activities of phytochemical resveratrol [27] and apigenin [32], UA was more effective in suppressing the cell viability in colorectal cancer cells (Figure 3). In addition, UA might be harmless to normal cells [21]. Thus, UA shows potential for colorectal cancer treatment.

Our TCF/β-catenin luciferase activity assay and analyses of the Wnt target gene expression through RT-qPCR and Western blotting demonstrated that UA can inhibit Wnt signaling (Figure 1 and Figure 2). The overexpression of Wnt signaling components and their inhibition by UA were used to identify the cellular targets of UA in the Wnt pathway. The overexpression of the dominant-active mutant of LRP5, namely LRP5△N, in the UA-treated P19 cells demonstrated that UA may exert its effects on LRP5 or some targets downstream of LRP5 (Figure 5A). Based on the assessment of the expression of Dishevelled-2, UA may target either Dishevelled or some protein downstream of Dishevelled in P19 cells (Figure 5B). Moreover, the treatment with LiCl (a GSK3β inhibitor) and UA indicated that UA may act on either GSK3β or some signaling components downstream of GSK3β (Figure 5C). The immunofluorescence staining for β-catenin in HCT-116 and Wnt-stimulated COS-7 cells demonstrated that compared with the vehicle, UA substantially reduced the levels of cytoplasmic and nuclear β-catenin (Figure 6). The Western blotting analyses of Wnt-stimulated P19 cells (Figure 1D and Figure 8A,B), Wnt-driven HCT-116 cells (Figure 2E and Figure 8C,D), SW480 cells (Figure 8E), and WiDr cells (Figure 8F) also demonstrated that UA reduced β-catenin protein levels. These data suggest that UA can regulate the protein stability of β-catenin under Wnt conditioning.

Three major degradation systems—namely the ubiquitin–proteasomal, autophagy–lysosomal [53], and calpain [54] degradation systems—regulate protein stability in cells. Of them, autophagy is a complicated cellular process, playing several pivotal roles: long-lived, aggregated, and misfolded protein removal; damaged organelle clearance; and cell growth and aging regulation [55]. In this study, the treatment of Wnt-stimulated P19 cells and Wnt-driven HCT-116 cells with the autophagy inhibitors chloroquine and wortmannin led to the recovery of UA-induced downregulation of β-catenin, with concomitant fluctuations of the autophagic markers LC3B and P62 (SQSTM1) (Figure 8A–D). The treatment of SW480 cells with wortmannin and that of WiDr cells with chloroquine to inhibit the autophagic influx compensated for the UA-elicited β-catenin degradation (Figure 8E,F). In addition, the treatment of Wnt-stimulated COS-7 cells and Wnt-driven HCT-116 cells with UA induced the punctate staining of the autophagy marker LC3B in the cytoplasm, indicating autophagosome formation (Figure 7). In summary, these results suggested that UA prompts the degradation of accumulated β-catenin both physiologically and pathologically through the autophagy–lysosomal degradation system under Wnt conditioning.

The PI3K/AKT/mTOR pathway is involved in autophagy [56]. Our results demonstrated that the treatment of HCT-116 cells with UA reduced the PI3 kinase, AKT, and mTOR activities. The reduction in mTOR activity results in the suppression of p70 S6 kinase and 4E-BP1, along with concomitant ULK1 activation (Figure 9), initiating the autophagy–lysosomal proteolysis system for the degradation of β-catenin and thus downregulating Wnt/β-catenin signaling.

Wnt/β-catenin signaling dysregulation occurs in many human diseases, including various types of cancers [57]. Targeting the central Wnt signaling component β-catenin may be an ideal approach to terminating activated Wnt signaling in these Wnt dysregulation-related diseases. However, targeting β-catenin with small-molecule inhibitors can be difficult [58]. Therefore, the development of newer methods to resolve this problem is warranted. For instance, proteolysis targeting chimera (PROTAC) is a well-designed technique for targeting protein degradation [36,59]. The PROTAC peptide xStAx-VHLL—where a stapled peptide xStAx of the β-catenin-binding site on Axin is fused with the Von Hippel–Lindau (VHL) ligand—can be used to target β-catenin for proteolysis. xStAx-VHLL effectively inhibits the Wnt signaling of cancer cells and APC^−/−^ organoids and reduces the tumor burden in BALB/c nude mice by targeting β-catenin for efficient degradation [60]. A sophisticated design and technique aid them in achieving the treatment goal. However, in this study, we used another approach to reduce β-catenin levels in cells, bypassing the need for a complicated design. Our results indicated that the treatment of cancer cells with the phytochemical UA can suppress Wnt/β-catenin signaling by reducing β-catenin levels through the autophagy–lysosomal degradation system via the PI3K/AKT/mTOR pathway inhibition. Our strategy is simple, easy, and low-cost for future clinical applications. Many neurodegenerative diseases, such as Parkinson’s disease, Alzheimer’s disease, Huntington’s disease, prion diseases, and amyotrophic lateral sclerosis, occur due to abnormal protein aggregation [61]. Our UA-elicited autophagy–lysosomal degradative system could eliminate pathological protein aggregates and thus alleviate these diseases.

Cancer stem cells, a subpopulation of cells with self-renewal and pluripotent differentiation capabilities, may be responsible for cancer recurrence, metastasis, and chemotherapy and radiotherapy resistance [62,63,64]. Wnt signaling is required for cancer stem cell maintenance [65]. Thus, drugs that can target Wnt/β-catenin signaling may solve the aforementioned issues related to cancer stem cells. Our data demonstrated that UA can inhibit Wnt activity (Figure 1A,C,D) and cell proliferation (Figure 3A) and delay the cell cycle progression (Figure 4A) in Wnt-stimulated P19 cells, which are embryonic carcinoma cells with stem cell properties differentiated into three germ layers on induction [66]. Therefore, UA is a promising phytochemical that can prevent cancer relapse, metastasis, and drug resistance by targeting Wnt/β-catenin signaling in cancer stem cells.

Aberrations in Wnt/β-catenin signaling cause tumorigenesis and metastasis [67], resulting in cancer development and progression. The phytochemical resveratrol can inhibit Wnt signaling by interfering with the association of TCF with β-catenin [27]. Here, we discovered that UA can downregulate β-catenin through the autophagy–lysosomal degradation system. Combination therapy is a promising strategy for treating diseases, including cancers [68,69]. Therefore, a combined treatment of UA with resveratrol may be more effective for cancer treatment; this is because resveratrol can eradicate residual β-catenin incompletely processed by the UA-elicited autophagy–lysosomal system.

**Figure 10:**
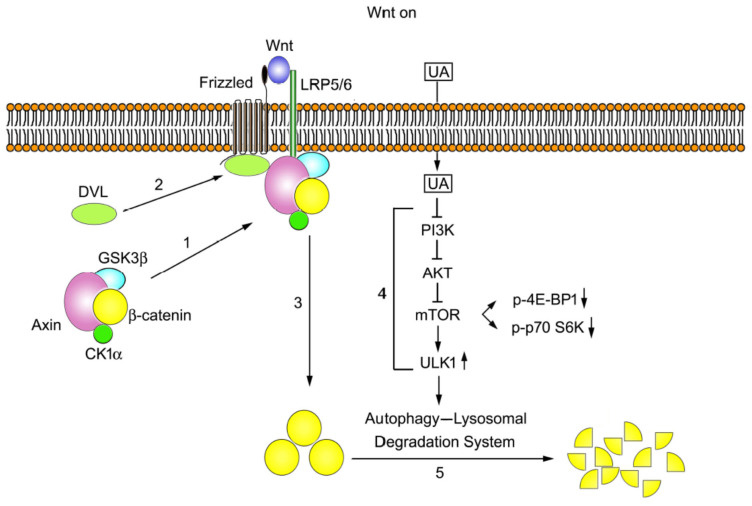
Molecular mechanisms underlying the UA-induced β-catenin downregulation in Wnt signaling. On the Wnt stimulation, the Axin complex (composed of Axin, β-catenin, GSK3β, and CKIα) moves from the cytoplasm to the cell membrane. Axin in this complex binds to phosphorylated LRP5/6, the Wnt coreceptor (Step 1). Dishevelled also moves to the cell membrane and binds with the Wnt receptor Frizzled (Step 2). β-catenin then becomes stabilized and accumulates in the cytoplasm (Step 3). UA reduces the PI3K, AKT, and mTOR activities consecutively, leading to the ULK1 activation and initiating autophagy (Step 4) and the subsequent degradation of β-catenin by the autophagy–lysosomal degradation system (Step 5). The decrease in the mTOR activity also results in the 4E-BP1 and p70 S6 kinase activity suppression.

Based on these findings, we propose a model to decipher the molecular mechanism underlying the effects of UA on Wnt signaling (Figure 10). Under Wnt stimulation, the Axin complex (comprising Axin, β-catenin, GSK3β, and CK1α) is transported from the cytoplasm toward the plasma membrane, and Axin in the complex binds to phosphorylated LRP5/6 (Step 1), whereas Dishevelled binds to Frizzled (Step 2). Next, β-catenin accumulates in the cytoplasm (Step 3). The treatment with UA can decrease the PI3K, AKT, and mTOR activities sequentially. A reduction in mTOR activity results in ULK1 activation to drive the autophagy–lysosomal degradation system (Step 4) for processing β-catenin into fragments for reuse through the anabolic pathway (Step 5). Moreover, the mTOR activity suppression leads to the inactivation of p70 S6 kinase and 4E-BP1, which are two downstream substrates of mTOR.

## 4. Materials and Methods

### 4.1. Materials

The antibodies (see Table 1 for details) against LC3B, Axin2, AKT, Phospho-AKT (Ser473), mTOR, Phospho-mTOR (Ser2448), p70 S6 kinase, Phospho-p70 S6 kinase (Thr389), 4E-BP1, Phospho-4E-BP1 (Ser65), ULK1, Phospho-ULK1 (Ser555), Phospho-ULK1 (Ser757), and Phospho-PI3 Kinase p85 (Tyr458)/p55 (Tyr199) were procured from Cell Signaling Technology (Danvers, MA, USA). The antibodies against GAPDH, SQSTM1 (p62), PI3-kinase p55γ, survivin, and c-Myc (9E10) were acquired from Santa Cruz Biotechnology (Dallas, TX, USA). An antibody against β-catenin was obtained from BD Transduction Laboratories (Franklin Lakes, NJ, USA). An antibody against Matrix metalloprotease 2 (MMP2) was procured from GeneTex (Alton Pkwy, Irvine, CA, USA), and the AlexaFluor 488 goat antirabbit IgG and AlexaFluor 546 goat antimouse IgG were obtained from Molecular Probes (Thermo Fisher Scientific, Waltham, MA, USA). The Peroxidase-conjugated AffiniPure goat antimouse IgG and Peroxidase-conjugated AffiniPure goat antirabbit IgG were acquired from Jackson ImmunoResearch Laboratories (West Grove, PA, USA). The 3-(4,5-Dimethyl-2-thiazolyl)-2,5-diphenyl-2H-tetrazolium bromide (MTT) was obtained from Sigma-Aldrich (St. Louis, MO, USA) and prepared in 1× phosphate-buffered saline (PBS). The UA was procured from Sigma-Aldrich (St. Louis, MO, USA) and dissolved in dimethyl sulfoxide (DMSO; Sigma-Aldrich, St. Louis, MO, USA). The Dual Luciferase Reporter Assay System was obtained from Promega (Madison, WI, USA). The Immobilon-P polyvinylidene fluoride (PVDF) membrane and Immobilon Western Chemiluminescent HRP Substrate were procured from Merck Millipore (Carrigtwohill, CO, USA). The EDTA-free protease inhibitor cocktail tablets and phosphatase inhibitor cocktail tablets were obtained from Roche (Mannheim, Germany). The 4′,6-Diamidino-2-phenylindole (DAPI) was procured from Invitrogen (Rockville, MD, USA). An AllPure Total RNA Isolation Kit was obtained from AllBio Science (Taichung, Taiwan). Finally, the SuperScript III First-Strand Synthesis System was obtained from Invitrogen (Rockville, MD, USA).

### 4.2. Cell Culture

The cells were maintained in a growth chamber under 5% CO_2_ and humidity and passaged at 2- or 3-day intervals, depending on the cell type. The WiDr, SW480, HCT-116, and COS-7 cells were cultured in Dulbecco’s modified Eagle medium (DMEM) containing 10% fetal bovine serum (FBS; Hyclone, Logan, UT, USA). The P19 cells were cultured in alpha-minimum essential medium containing 7.5% bovine calf serum (Hyclone) and 2.5% FBS. The control-conditioned and Wnt-3a-conditioned media were prepared as described previously [27,28]. All cells were purchased from the cell bank of Bioresource Collection and Research Center (Hsinchu, Taiwan).

### 4.3. Western Blotting

The cells were suspended in cell lysis buffer (1% Triton X-100; 5 mM EDTA; 50 mM Tri-HCl, pH 8.0; 150 mM NaCl; and protease and phosphatase inhibitors), followed by incubation on a rotatory shaker at 4 °C for 10 min. Next, the lysed cells were centrifuged at 12,000 rpm at 4 °C for 15 min. The supernatant was transferred into a new microcentrifuge tube and was regarded as the total cell lysate. The total cell lysate was quantified using a Protein Assay Dye Reagent (Bio-Rad, Hercules, CA, USA). Equal amounts of proteins were mixed with a Laemmli sample buffer, boiled for 5 min, and fractionated through sodium dodecyl sulfate polyacrylamide gel electrophoresis. Proteins on the gel were electroblotted onto PVDF membranes, and proteins on these membranes were detected through enhanced chemiluminescence, as described previously [27,28,32]. The images were captured by the ChemiDoc XRS+ imaging system equipped with Image Lab^TM^ software version 6.0.0. (Bio-Rad Laboratories, Inc.).

### 4.4. Reverse Transcription Quantitative Polymerase Chain Reaction

The cells were treated and collected for the isolation of total RNAs by using the AllPure Total RNA Isolation Kit. Total RNAs were quantified, and equal amounts of total RNAs were converted into cDNA by using the SuperScript III First-Strand Synthesis System. Thereafter, the cDNAs were subjected to quantitative polymerase chain reaction (qPCR) using a 2× master mix from the KAPA SYBR FAST qPCR Kit (Kapa Biosystems, Wilmington, MA, USA), as described previously [27]. The primer sequences used here were as described previously [32].

### 4.5. Immunofluorescence Staining

The cells were seeded onto coverslips in a culture dish, incubated for 1 day, and then treated with UA for the indicated period. The culture medium was removed, and the cells were washed three times with 1× PBS for 10 min each time. The cells were then fixed with 4% paraformaldehyde at room temperature for 15 min, washed with 1× PBS, and then permeabilized with 0.1% Triton X-100 in 1× PBS. For the autophagosome detection, the cells were further permeabilized with ice-cold methanol for 10 min. Thereafter, the cells were washed extensively with 1× PBS and incubated with a primary antibody at 4 °C overnight. The cells were then washed extensively with 1× PBS and incubated with fluorophore-conjugated secondary antibodies at room temperature for 1 h in the dark. The cells were then stained with 5 mg/mL DAPI (1:1500) for 10 min, washed with 1× PBS, and mounted onto a prelabeled slide using a water-based mount medium (Electron Microscopy Sciences, Hatfield, PA, USA). The slide was then air-dried in the dark, followed by the data collection under a fluorescent microscope [27,32].

### 4.6. Dual Luciferase Activity Assay

The cells (1 × 10^5^/well) were seeded into each well of a 24-well dish and cultured overnight for attachment. The cells were transfected with the Wnt reporter plasmid pGL3-OT, or pTOPFlash (for WiDr cells), the normalization plasmid pTK-Renilla, and/or effector plasmids by using LipoFectamine 2000 (Thermo Fisher Scientific) for 8 h. The cells were then treated with UA for the indicated periods. Next, the medium was removed, and the cells were washed. The cells were then lysed with 1× passive lysis buffer, followed by incubation at room temperature for 20 min on a rotatory shaker. The cell lysates were collected for dual luciferase activity measurement, and the obtained data were further processed in MS Excel to calculate the relative Wnt reporter activity between treatments [27,28]. For each treatment, three replicates were used.

### 4.7. Cell Viability Assay

Here, the MTT assay was used for evaluating the cell viability [27,32]. The cells (2 × 10^3^/well) were seeded into each well of a 96-well dish and cultured overnight for attachment. The cells were then treated with the indicated drugs and incubated for an additional 96 h. Next, 100 µL of the culture medium was removed, and 50 µL of MTT (2 mg/mL) was added to the wells. The cells were then incubated until formazan crystals formed. Next, the cells were centrifuged at 2500 rpm for 20 min, and the excess MTT in the supernatant was removed. Subsequently, to dissolve the formazan crystals, 100 µL of DMSO was added to the cells, followed by shaking for 15 min in the dark. Finally, the absorbance at 570 nm was measured on an enzyme-linked immunosorbent assay reader (Thermo Fisher).

### 4.8. Cell Cycle Analysis Through Flow Cytometry

The P19 cells were treated with UA at different concentrations in a Wnt-3a-conditioned medium for 16 h, whereas the colorectal cancer cells were treated with UA at different concentrations for 22 h. Next, the cells were trypsinized, centrifuged, collected, and resuspended at 2 × 10^6^/mL in 1× PBS. After three washes in ice-cold 1× PBS, the cells were resuspended in 1.5 mL of cold 1× PBS. Next, to fix the cells, 3.5 mL of ice-cold ethanol was added dropwise to the cells with slow vortexing. Thereafter, the treated cells could be incubated at −20 °C until further analysis.

In the next step, the cells were washed three times with 1× PBS and stained with propidium iodide (PI). The cell cycle distribution was analyzed on a fluorescence-activated cell sorter (BD FACS Canto, Franklin Lakes, NJ, USA) [70].

### 4.9. Statistics

The statistical analysis was performed using a paired two-tailed Student’s *t*-test using GraphPad Prism version 5.0 software. The data represent the mean ± standard deviation (SD). The differences were considered statistically significant as *p* < 0.05 (* *p* < 0.05, ** *p* < 0.01, *** *p* < 0.001).

## 5. Conclusions

In conclusion, our results demonstrated that UA can inhibit cell growth and cell cycle progression in Wnt-stimulated P19 cells and colorectal cancer cells by targeting β-catenin for autophagy–lysosomal degradation through the PI3K/AKT/mTOR pathway inhibition. Thus, UA demonstrates therapeutic potential for Wnt-related diseases with abnormally accumulated β-catenin, such as Wnt-driven colorectal cancer; it can also be a promising therapeutic agent for neurodegenerative diseases due to protein aggregation.

## Figures and Tables

**Table 1:** Detailed information regarding the antibodies used in this study.

Name	Species	Dilution	Application	Catalogue Number	Brand Name
AKT (pan) (C67E7)	Rabbit mab ^a^	1:1000	WB ^b^	#4691	Cell Signaling Technology
Phospho-AKT (Ser473) (D9E) XP	Rabbit mab	1:2000	WB	#4060	Cell Signaling Technology
Axin2 (76G6)	Rabbit mab	1:1000	WB	#2151	Cell Signaling Technology
LC3B	Rabbit	1:1000	WB; immune ^c^	#2775	Cell Signaling Technology
mTOR	Rabbit mab	1:1000	WB	#2972	Cell Signaling Technology
Phospho-mTOR (Ser2448)	Rabbit	1:1000	WB	#2971	Cell Signaling Technology
p70 S6 kinase	Rabbit mab	1:1000	WB	#9202	Cell Signaling Technology
Phospho-p70 S6 kinase (Thr389)	Rabbit mab	1:1000	WB	#9205	Cell Signaling Technology
4E-BP1 (53H11)	Rabbit mab	1:1000	WB	#9644	Cell Signaling Technology
Phospho-4E-BP1 (Ser65)	Rabbit	1:1000	WB	#9451	Cell Signaling Technology
ULK1 (D8H5)	Rabbit mab	1:1000	WB	#8054	Cell Signaling Technology
Phospho-ULK1 (Ser555) (D1H4)	Rabbit mab	1:1000	WB	#5869	Cell Signaling Technology
Phospho-ULK1 (Ser757) (D1H4)	Rabbit	1:1000	WB	#6888	Cell Signaling Technology
Phospho-PI3 Kinase p85 (Tyr458/p55Tyr199)	Rabbit	1:1000	WB	#4228	Cell Signaling Technology
GAPDH (6C5)	Mouse mab	1:3000	WB	sc-32233	Santa Cruz Biotechnology
SQSTM1 (D-3)	Mouse mab	1:500	WB	sc-28359	Santa Cruz Biotechnology
PI3-Kinase p55γ (E-9)	Mouse mab	1:500	WB	sc-376615	Santa Cruz Biotechnology
Survivin (D-8)	Mouse mab	1:500	WB	sc-17779	Santa Cruz Biotechnology
c-Myc (9E10)	Mouse mab	1:500	WB	sc-40	Santa Cruz Biotechnology
β-catenin	Mouse mab	1:1000	WB; immune	610154	BD Transduction Laboratories
MMP2	rabbit	1:1000	WB	GTX104577	GeneTex

^a^ mab: monoclonal antibody; ^b^ WB: Western blotting; ^c^ immune: immunostaining.

## Data Availability

The original contributions presented in this study are included in the article. Further inquiries can be directed to the corresponding author.

## Abbreviations

AKT, protein kinase B; APC, Adenomatous Polyposis Coli; AMPK, AMP-activated protein kinase; LC3B, microtubule-associated protein 1 light chain 3 beta; ULK1, uncoordinated-51-like kinase 1; PI3K, phosphatidylinositol 3-kinase, PI3P, phosphatidylinositol 3-phosphate; EGFR, epidermal growth factor receptor; mTOR, mammalian target of rapamycin; VEGFR, vascular endothelial growth factor receptor; ROS, reactive oxygen species; PE, phosphatidylethanolamine; RT-qPCR, quantitative reverse transcription PCR; CRC, colorectal cancer.
